# Docking-Based Classification of SGLT2 Inhibitors

**DOI:** 10.3390/molecules30102179

**Published:** 2025-05-16

**Authors:** Ajouan Mazoudji, Gerhard F. Ecker

**Affiliations:** Department of Pharmaceutical Sciences, University of Vienna, Josef-Holaubek-Platz 2 (UZA II), 1090 Vienna, Austria; ajouan.mazoudji@univie.ac.at

**Keywords:** docking, SGLT2, SLC5A2, diabetes, classification models

## Abstract

Inhibitors of the Sodium/Glucose co-transporter 2 (SGLT2) have been evolving into an important contribution to the treatment of diabetes mellitus. As the inhibition of SGLT2 is sensitive to the structural configuration at the sugar moiety of the inhibitors, it is of high interest to provide in silico-based methods for the prediction of the activity of potential SGLT2 inhibitors that take three-dimensional information into account. To attain this objective, a classification model based on the docking scores obtained from the best-performing docking-based virtual screening was created. Furthermore, the impact of ensemble docking using docking results from five SGLT2 structures and the incorporation of structural similarity information was assessed by creating classification models using these approaches. Taking a combined approach of docking score and structural similarity modelling led to the best performance with a Matthews Correlation Coefficient (MCC) of 0.64. Finally, to explore the ability of the used docking algorithms to correctly predict the influence of different three-dimensional information, a library of molecules with a negatively contributing configuration was created and docked, showing decreased docking scores for the molecule library with a disadvantaged configuration.

## 1. Introduction

Diabetes mellitus represents one of the most significant global health emergencies of the 21st century and is a serious chronic condition. Numbers from 2021 estimate that 537 million adults live with diabetes, representing 10.5% of the world’s adult population. This number is expected to further increase and reach 643 million people by 2030 [1]. In spite of the existence of a diverse number of therapeutic options for the treatment of type 2 diabetes, which is the most common type, with 90% of the cases being classified as type 2 diabetes, a significant number of people are affected by failure in glycaemic control, which can lead to adverse effects such as weight gain and hypoglycaemia [1,2].

Furthermore, the majority of existing treatments for type 2 diabetes are dependent on the production of insulin, which might, therefore, require an increase in the dose while the disease advances and insulin production declines [3]. For this reason, it has become apparent that new therapeutics with reduced undesirable side effects and no insulin dependence are needed in order to maximise the patient’s quality of life.

Sodium/Glucose co-transporter 2 (SGLT2) inhibitors are a drug family that has been recently introduced onto the market, where they have been mainly used as second-line therapeutics for the treatment of type 2 diabetes. They show a unique mechanism of action that allows them to exert the above-mentioned properties of avoiding insulin dependence and having reduced undesirable side effects [2]. The history of SGLT2 inhibitors can be traced back to more than a hundred years ago, when the natural compound phlorizin was isolated from the bark of the apple tree and identified to have a glycosuric effect [4]. However, its low selectivity for SGLT2, leading to frequent gastrointestinal side effects, and its degradation by glucosidase in the intestine prevented the clinical use of phlorizin [4]. The process of searching for potent and selective SGLT2 inhibitors, which are not degraded in the intestine, resulted in the development of compounds that were recently introduced onto the market, among them dapagliflozin, canagliflozin, and empagliflozin. These inhibitors are characterised by a glucose nucleus, a C-glycosidic moiety at position C1 of the sugar, and two aromatic rings connected by a methylene bridge [5,6].

**Figure 1:**
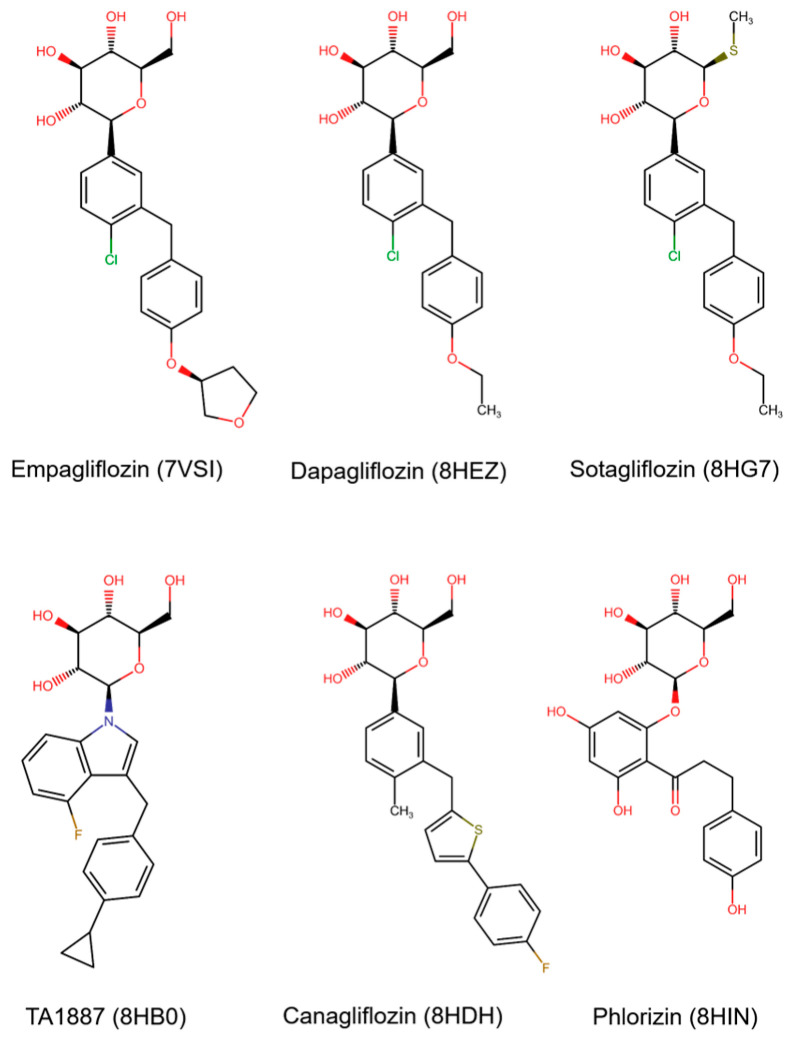
Ligands of SGLT2 with the PDB IDs of the structures they have been resolved with. The synthetic gliflozins empagliflozin, dapagliflozin, sotagliflozin, TA1887, and canagliflozin have been resolved with SGLT2 in an outward-open conformation, while the natural product phlorizin was resolved with SGLT2 in an inward-open conformation.

**Figure 2:**
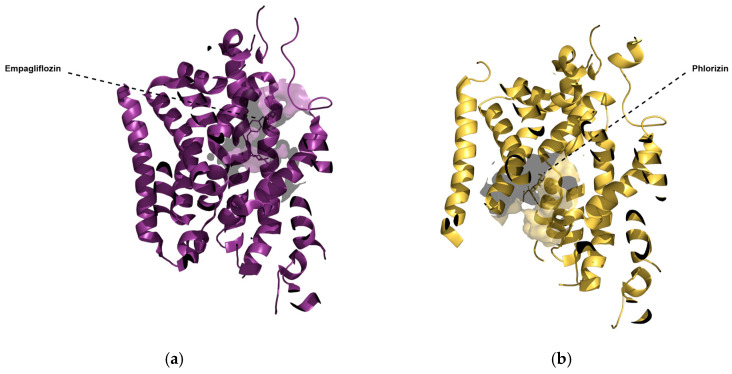
Comparison of (**a**) one of the outward-open structures of SGLT2 with empagliflozin bound to it and (**b**) the inward-open structure with phlorizin bound to it. The binding sites of both structures have been visualised as shadows, and the two structures were aligned to make a direct comparison possible. The binding site occupied by phlorizin in the inward-open conformation as well as the helices with which it interacts are distinct from the binding site occupied by empagliflozin in the outward-open conformation.

In 2021, a cryogenic electron microscopy structure of SGLT2 with empagliflozin at its binding site was published for the first time [7]. The subsequent publication of four additional SGLT2 structures in their outward-open conformation and one SGLT2 structure in its inward-open conformation [8] added vital information about its transport and inhibition mechanism. All of the six above-mentioned structures were resolved with a ligand at their binding site, the structures of which are displayed in Figure 1. Except for phlorizin, which is a natural product and can bind to SGLT2 in its inward-open conformation (PDB ID: 8HIN), all five bound ligands are synthetic gliflozins that exclusively bind to SGLT2 in its outward-open conformation [7,8]. As shown in Figure 1, synthetic gliflozins usually possess a sugar moiety, which is typically a hexose, since changing the ring to a pentose may lead to loss of activity [5]. Connected to the sugar at position C1 is an aglycone group with hydrophobic properties, usually consisting of two aromatic rings connected to each other through a methylene bridge [5]. The direct connection of the central aromatic ring of synthetic gliflozins to the glucose moiety leads to a rigidity that prevents binding to the inward-open structure. In contrast to the above-mentioned direct connection, phlorizin possesses an oxygen bridge connecting the central aromatic ring with the aglycone group, leading to the possibility of inward-open binding. The differences between the two binding modes and the two conformations are presented in Figure 2. As shown in Figure 2, the binding site of the inward-open conformation is distinct from the binding site of the outward-open conformation, with phlorizin mainly interacting with different helices (TM1, TM5, and TM8) than synthetic gliflozins (TM1, TM2, TM3, TM6, and TM10) [8].

As mentioned above, despite multiple existing therapies, the need for novel molecules with reduced side effects for the treatment of type 2 diabetes persists. However, the introduction of new drugs to the market is a tedious and expensive process over the course of 10–15 years [9,10]. To improve the costs and efficiency of tasks involved in the preliminary stage of drug discovery, Computer-Aided Drug Discovery (CADD) has become an important part of the process [9]. One of the main areas within the CADD domain is virtual screening, which is the application of computational tools to search large databases for new lead molecules with a strong binding affinity to the target. Virtual screening approaches can usually be classified into structure-based approaches, where the structure of the protein target is known, and ligand-based approaches, where only ligand structures are known [11]. Among the structure-based approaches, molecular docking is one of the most often used tools in computer-aided drug design and has been successfully deployed for the development and improvement of several drugs [12]. It is a widely used computational tool for studying the process of molecular recognition with the aim of predicting the binding mode and affinity of a molecule resulting from the establishment of a complex with its target [13]. The main approach used among the ligand-based virtual screening methods is Quantitative Structure-Activity Relationship (QSAR). QSAR is a popular ligand-based approach that is employed in order to correlate chemical molecules with their biological and pharmaceutical activities based on their chemical structure [14,15] by calculating mathematical descriptors that encode molecular structures and properties [15,16].

While a number of QSAR regression model studies based on 2D descriptors have been conducted for SGLT2 inhibition [17,18], no such case is known for classification models. Additionally, QSAR studies resulting in regression models based on 3D descriptors have only been performed with limited datasets [19,20]. As shown by various SAR explorations of SGLT2 inhibitors [5], the three-dimensional properties, namely the configuration of the positions C1 and C5 of the sugar moiety, are known to substantially impact the activity of SGLT2 inhibitors. The reversion of the configuration at these positions can decrease the activity of SGLT2 inhibitors and even lead to abolishing their activity entirely [5]. Therefore, it is of special interest to take docking-based in silico methodologies, which are able to discern between different configurations, into account to support the development of novel SGLT2 inhibitors.

Furthermore, QSAR approaches are only able to ensure reliable predictions for chemicals similar to those already known, while docking-based models have the advantage of being able to employ the physicochemical information of the binding pocket to enlarge the applicability domain beyond the boundaries of QSAR models [21]. Accounting for the lack of classification models for SGLT2 and the inability of 2D QSAR models to incorporate three-dimensional information into their algorithms, a necessity for providing a docking-based framework for the classification of SGLT2 inhibitors has been identified.

With this background in mind, the aim of this work is to provide three-dimensionally aware, well-performing in silico-based methods, mainly driven by the use of molecular docking, for the assessment of the activity of potential SGLT2 inhibitors that have not yet been tested in vitro. For this reason, multiple docking-based classification approaches have been explored, resulting in a range of classification models for the prediction of the activity of potential SGLT2 inhibitors.

The approaches of this study for creating docking-supported classification models comprise three main methodologies.

Firstly, a docking score-based approach was taken to create a classification model, which categorises any molecule above or below a certain docking score threshold either into a positive or negative category by using docking scores resulting from the best-performing SGLT2 structure. Similar approaches have been successfully employed for endocrinal disruption [21,22] and inhibitors of P-glycoprotein [23].

Secondly, the effect of combining results from docking into multiple SGLT2 structures to improve upon the aforementioned docking score-based approach was investigated. This method is known as ensemble docking and is well-established in the field of early-stage drug discovery. Studies that have been performed as early as 1999 have been able to show that using multiple protein structures for binding predictions can be more successful than models based on single conformations alone [24,25]. We, therefore, made use of the multiple protein structures available for SGLT2 to explore the effects of ensemble docking on the performance of the resulting classification models.

Finally, the effects of using a recently developed method for improving scoring functions by incorporating structural similarity to ligands with known activity were explored [26]. The method, which introduces an algorithm for correcting docking scores based on the scores of structurally similar molecules, is a promising concept that combines the known activities of ligands (similar to QSAR methods) and the advantages of structure-based approaches.

In addition to the above-mentioned methods for the creation of classification models, an analysis of the effect of incorporating 3D information from docking studies into classification models has been conducted. To analyse the consequences of a changed configuration at position C5 of SGLT2 inhibitors, one of the developed methods was applied to a dataset of SGLT2 inhibitors and their counterparts with a changed configuration at position C5. The analysis was not only performed to assess the abilities of the introduced methods but also provided valuable information about the reasons for the loss of activity of molecules with an *S*-configuration at position C5.

The resulting classification models showed a high performance and displayed the ability to discern between molecules that only differ in their configuration at position C5 of the sugar moiety. Out of the multiple created classification models, the similarity-based models achieved the best performance, while ensemble docking models outperformed the docking score-based models.

## 2. Results and Discussion

### 2.1. Suitability Analyses

Five protein structures of human SGLT2 with an outward-open conformation from the Protein Data Bank (PDB) and 1197 ligands with known activity values from ChEMBL were used to perform docking studies. The suitability of the chosen SGLT2 structures for our study was judged by redocking their ligands, and the performance of the chosen docking protocol was assessed by screening a library of compounds with known activity collected from ChEMBL. The results from the docking studies were then assessed by calculating the 10% Enrichment Factor (EF) and the Receiver Operator Characteristics Area Under the Curve (ROC AUC) for each of the protein structures. All the SGLT2 structures were able to reproduce the pose of their bound ligand in the redocking studies, and only a low number of ligands could not be docked into each of the protein structures, with PDB ID 8HB0 showing the highest amount (85), and PDB ID 8HG7 the lowest amount (39). The redocked and cryo-EM poses of the bound ligands are provided in the Supporting Information Figure S1. Out of the five analysed SGLT2 structures, PDB ID 7VSI displayed the best performance, as presented in Table 1, with a ROC AUC of 0.72 and a 10% EF of 1.5. The results showed the ability of the docking protocols to correctly reproduce the poses of the ligands and to achieve an enrichment of the active molecules, which proved the usability of the chosen docking protocols and protein structures for the aims of this study.

### 2.2. Docking Score-Based Classification

**Figure 3:**
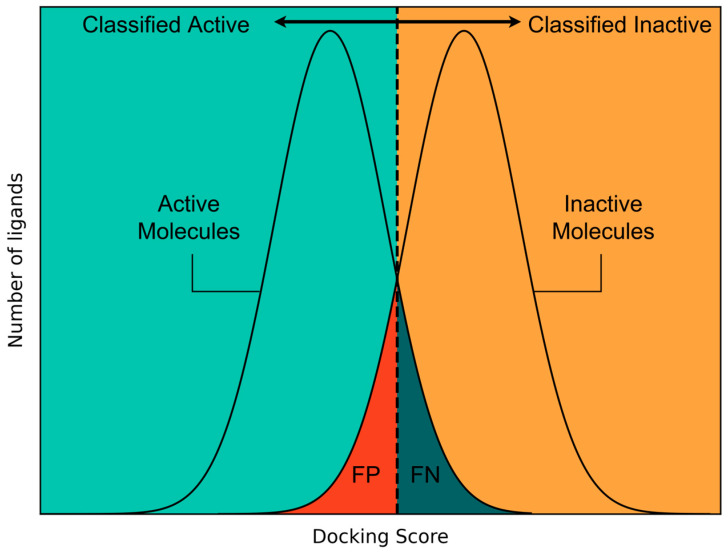
Calculation of a threshold for the creation of docking score-based classification models. The threshold is chosen by identifying the intersection of the distributions of the active (left curve) and inactive (right curve) compounds. Any compound to the right of the intersection will be classified as inactive, while any compound to the left will be classified as active. Compounds that belong to the active curve and are located to the right of the threshold are False Negatives (FN), while compounds that belong to the inactive curve and are located to the left of the threshold are False Positives (FP).

Based on the results of the docking studies, it was decided to use the docking scores provided by docking into 7VSI to create a classification model since it showed the best performance in the virtual screening validation. The classification model was generated by creating a distribution function based on docking scores for the active and inactive molecules and calculating the intersection point of the two curves. This results in a threshold for the classification of docked molecules into an active or inactive category. Compounds with a higher docking score than this threshold were classified as inhibitors of SGLT2, while compounds with lower docking scores were considered non-inhibitors. Figure 3 shows an example of the chosen approach for identifying a threshold for classification models. Additionally, a detailed description of this method can be found in Section 3.3. This method for identifying a score threshold for the classification of molecules has been used throughout this study for all three explored classification approaches (docking score-based classification, ensemble docking-based classification, and structural similarity-based classification) and will be referred to as the “Threshold Method”.

**Figure 4:**
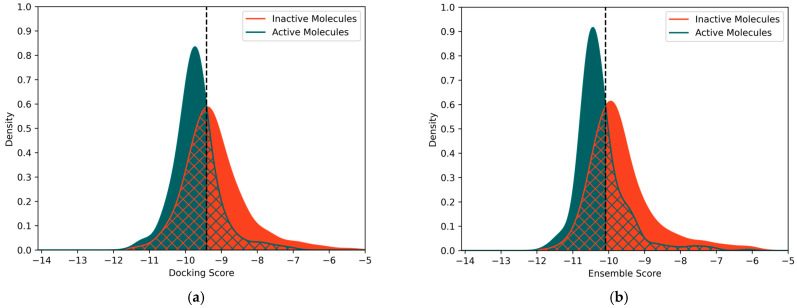
Comparison of the distributions of the active (green) and inactive (orange) compounds using (**a**) 7VSI and (**b**) 7VSI, 8HEZ, and 8HG7. The fully coloured areas correspond to the correctly classified molecules, while the mesh corresponds to false positive (orange) and false negative (green) instances. The intersections resulting from these distributions were used to create the docking score- and ensemble score-based classification models presented above by employing the threshold method introduced in Section 2.2.

The resulting classification model based on docking with 7VSI was derived from an intersection point of −9.41 of the distribution curves, as displayed in Figure 4a. The performance of the model was assessed by applying it to the dataset it was derived from, which showed a Matthews Correlation Coefficient (MCC) of 0.36 and a Balanced Accuracy (BA) of 0.68.

### 2.3. Ensemble Docking-Based Classification

In order to explore the possibility of using docking scores originating from multiple structures of the same transporter, an ensemble docking-based approach was taken. In ensemble docking, candidate ligands are docked into an “ensemble” of conformations of the drug target [24]. The existence of five SGLT2 structures with slightly differing conformations, therefore, allowed an investigation of the usefulness of ensemble docking for potential SGLT2 inhibitors. Ensemble scores were calculated by looping through all possible combinations of the available SGLT2 structures with varying lengths (2–5) and calculating the mean of the docking scores obtained from these structures. A total of 26 ensemble docking-based classification models were generated (Table S1) by applying the threshold method described in Section 2.2 and Section 3.3.

Using the above-mentioned ensemble docking-based approach led to significant improvements in the resulting classification models, as analysed by applying them to the datasets they were derived from. This was especially the case for the model based on the structures 7VSI, 8HEZ, and 8HG7, where an MCC of 0.41 and a BA of 0.71 were obtained, while using all five structures barely further improved the model, leading to an MCC of 0.36 and a BA of 0.68. The best-performing model from each combinatory length is shown in Table 2. The improvement of the performance of the classification model when using ensemble docking is displayed in Figure 4, which shows the distributions of active and inactive molecules according to their docking scores (Figure 4a) and their ensemble scores (Figure 4b). The distributions for the ensemble score show a higher number of active molecules located on the left side of the classification threshold, leading to a higher number of correctly classified instances of active molecules. Additionally, a shift in the distribution function of inactive molecules to the right relative to the distribution function of active molecules can be seen. This difference in the position of the distribution functions leads to covering a higher number of inactive molecules when classifying molecules to the right of the intersection as inactive. This results in a better discrimination between active and inactive molecules and has been reflected in a clearly improved performance of the classification model based on 7VSI, 8HEZ, and 8HG7.

### 2.4. Structural Similarity-Based Classification

Furthermore, the usability of a recently introduced method for enhancing scoring functions [26], was explored. This methodology introduces a correction algorithm for docking scores using structural similarity and the difference between docking scores and known molecular activities. The Tanimoto similarity between each molecule in the query dataset and those in a reference dataset with known binding affinities and docking scores are calculated. The calibration of the query dataset’s docking scores is then based on the weighted differences between the docking scores and experimental binding affinities of the reference molecules, where weights are determined by structural similarity. Molecules with higher similarity to a query molecule exert a stronger influence on its calibration. This similarity-based method was adapted to examine its applicability to classification tasks for SGLT2 inhibitors. Ultimately, the approach was improved upon by exploring a wider range of values for exponent *p*, which is a variable that determines the importance placed upon the similarity of a query molecule to the reference dataset molecules. Additionally, three alternative fingerprint algorithms for the similarity calculations were tested and compared. Additional information regarding the used algorithm is presented in Section 3.3.

**Figure 5:**
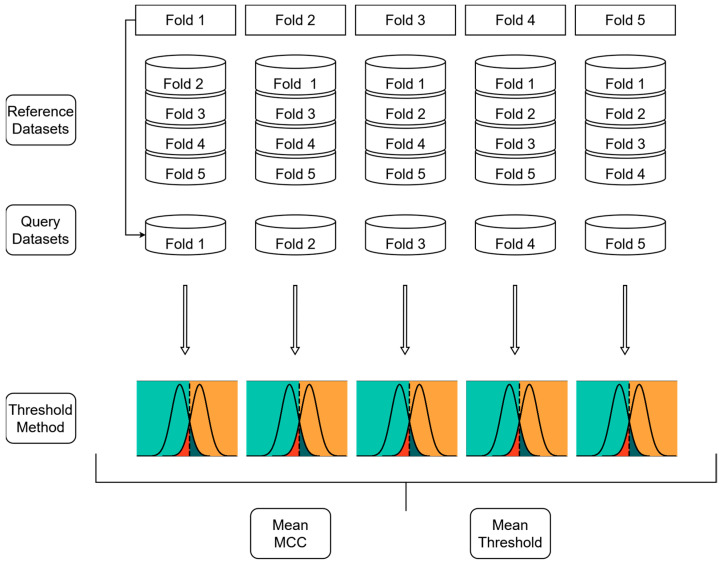
Approach for the creation of similarity-enhanced classification models. A five-fold cross-validation has been used, with the final step corresponding to the threshold method described in Figure 3. This approach was applied for each exponent *p*, which were then compared to each other by calculating the mean MCC across the five query sets. The mean threshold of the best-performing value for exponent *p* was then used as the final classification threshold. This was conducted for all three fingerprint types.

Since the similarity-based scoring function can only be applied to a subset of the dataset (referred to as the “query dataset”) [26], a five-fold cross-validation approach was implemented. As described in Figure 5, the dataset obtained from docking into 7VSI was first divided into five folds using a stratified 5-fold splitting method. Each of these five folds was treated as a query dataset once and subjected to the similarity-based algorithm to generate enhanced docking scores.

In the next step, each query dataset was used as input for classification models, which were built by identifying the intersection points of active and inactive molecule distributions. To evaluate the impact of different values of exponent *p*, a grid search was performed, and the mean MCC across all five query datasets was calculated to compare the different values of exponent *p*. Similarly, the threshold values for classification were determined by calculating the mean threshold across all five query datasets. These averaged thresholds were then used to apply the final classification models from each fingerprinting method to an external dataset, ensuring the generalisability of the approach.

Among all fingerprints and exponent *p*-values, the Morgan fingerprint with an exponent *p* of 12 achieved the highest MCC of 0.64 in the internal five-fold cross-validation of the classification models, which represents a significant improvement in performance compared to the docking score- and ensemble docking-based models.

**Figure 6:**
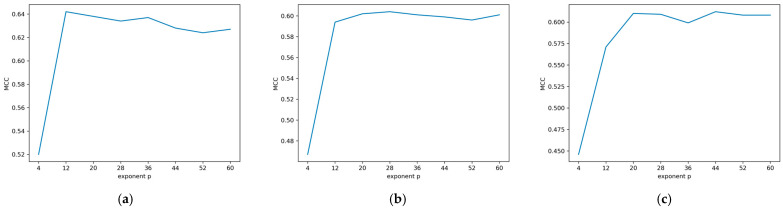
Matthews correlation of (**a**) Morgan fingerprint (**b**) RDKit fingerprint, and (**c**) MACCS keys models for exponent *p*-values ranging from 4 to 60 in steps of 8. The fingerprints showed their best performance at exponent *p*-values of 12, 28, and 44, with the Morgan fingerprint achieving the best performance among all fingerprints with an MCC of 0.64.

The performance of the various exponent *p*-values for each of the three fingerprint methods can be seen in Figure 6 and Table S2, where a higher exponent *p* does not necessarily lead to a higher MCC. This indicates an ideally balanced contribution of the docking scores and the structural similarity to the resulting classification model at an exponent *p* of 12 for the Morgan fingerprint. This supports the value given to the docking scores in this study, as the model does not improve—and even worsens—after a certain exponent *p*-value, where a higher exponent *p* represents a higher importance given to the structural similarity. Performances of the best classification model from each fingerprint method in the cross-validation, when tested against an external dataset, and their associated exponent *p*-values and intersection points are provided in Table 3. Even though the original docking scores possess negative values and describe the binding affinity of the molecules to their target, the scores resulting from the calibration with this methodology are positive, as they represent an approximation of the pChEMBL values. The average intersection points resulting from the cross-validation (7.82, 7.82, and 8.04) are very close to the pChEMBL value of 8 that was chosen as a threshold for classifying the molecules into active and inactive categories. The proximity of the intersection points to the real-world threshold further supports the usage of the proposed methodology, showing that the calibrated scores can reach values close to the pChEMBL values derived from experimental studies.

Consequently, it was deduced that the structural similarity-based classification methodology shows the best performance among all three studied methods, while the ensemble docking method outperforms the docking score method. A direct comparison of the best-performing model from each of the docking score-, ensemble score-, and structural similarity-based classification algorithms is shown in Table 4.

### 2.5. Three-Dimensional Influence

The beneficial influence and high performance of using docking-based approaches for the prediction of SGLT2 inhibitors can be explained through the incorporation of three-dimensional information into the scoring algorithms. SGLT2 seems to be sensitive to configurations of the sugar moiety, as shown by various SAR explorations of SGLT2 inhibitors [5].

**Figure 7:**
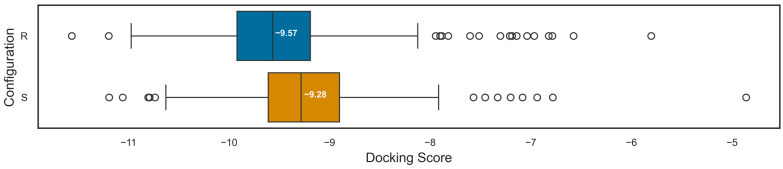
Box plots and medians of the *R*- (blue) and *S*-configuration libraries (orange). The medians of the *R*- (−9.57) and *S*-configuration (−9.28) correspond to the difference in the means, and the visual representation shows a shift in the *S*-configuration to the right, indicating an overall lower docking score for *S*-configuration molecules.

To further explore the ability of docking algorithms to correctly predict the influence of different three-dimensional information, a library of 314 molecules with *R*-configuration at position C5 of the sugar moiety was compared to a library of identical molecules with *S*-configuration at position C5 by docking both libraries into 7VSI. Molecules with *S*-configuration at this position are known to display a 300-fold decrease in activity [27], which is reflected in the results, where the *S*-configuration library had a mean docking score of −9.24 as opposed to −9.50 for the *R*-configuration library and a median docking score of −9.28 as opposed to −9.57. Out of 314 molecules of the *R*-configuration library, 302 were successfully docked into 7VSI, while 297 molecules of the *S*-configuration library were docked. The differences in the docking scores have been visually and statistically analysed. To visualise the calculated differences, box plots of the *R*- and *S*-configuration were created (Figure 7) and compared, clearly showing an overall shift in the docking scores of the *S*-configuration, and therefore indicating a lower binding affinity. In order to analyse the significance of the observed difference between the mean docking scores, a paired samples *t*-test has been conducted using the docking results of the two libraries. As mentioned above, a small portion of both libraries could not be docked, with 291 molecules being present in the docking results of both libraries. Therefore, the *t*-test was conducted using these 291 molecules. The null hypothesis was defined as a lack of true mean difference between the docking scores of the two libraries, and the significance level was set to 5%. The *t*-test resulted in a *p*-value of $6.91\times 10^{-12}$ with a 95% confidence interval of [−0.34, −0.19], allowing for the rejection of the null hypothesis. This is additionally supported by applying the classification model for 7VSI, which has a threshold of −9.41, classifying the mean score of the *S*-configuration library as inactive and the mean score of the *R*-configuration library as active.

**Figure 8:**
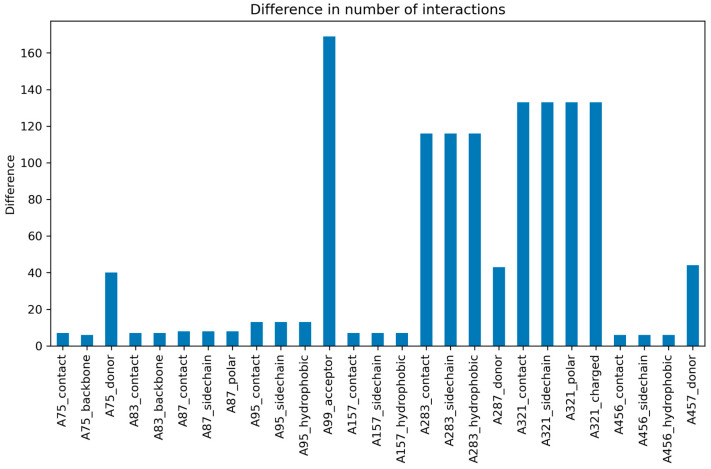
Differences in the number of amino acid interactions between the two libraries for amino acid interaction types with more than five lost interactions, visualised as a bar chart. The output of the interaction fingerprint panel does not specify the amino acid types. They can, however, be derived from the numbering system that is included in the output. The most significant interaction losses for the *S*-configuration library included the amino acids Glu99, Lys321, and Leu283.

Poses of the docked molecules from both libraries were compared to each other by calculating the Structural Interaction Fingerprint, which is a 1D binary fingerprint of structural binding information of a ligand-target complex [28], for each of the docked ligands using the Interaction Fingerprint Panel from the Schrödinger Software Suite. For each of the interaction types for every amino acid, the sum of the interacting ligands was calculated. Thereafter, the differences between the sums from the *S*-configuration library and the *R*-configuration library were calculated for each amino acid interaction type. As can be seen in Figure 8, amino acid interactions that were reduced by the changed configuration were then visualised as a bar chart in order to identify amino acids that are pivotal for activity loss in the *S*-configuration library.

The amino acid interaction which showed the strongest difference between the two libraries was the hydrogen bond accepting interaction of amino acid Glu99 with 169 ligands losing this interaction, making up 53.8% of the docked library. This is followed by various interaction types of the amino acids Lys321 with 133 lost interactions (42.4%) and Leu283 with 116 lost interactions (36.9%). Additionally, Asn75, Ser287, and Gln457, which are known to be hydrogen bond donors in SGLT2 inhibition [29], show interaction losses in some cases.

Out of the three amino acids with the highest interaction losses, Glu99 proved to be of high importance, as it serves as a hydrogen acceptor in the ligand binding process. The significance of the conserved residue Glu99 has been corroborated by a molecular dynamics investigation of drug dissociation from SGLT2 by Pang et al. [29], showing it to be critical for hydrogen bonding and contributing to hydrophobic interactions of selective SGLT2 inhibitors with their targets. Additionally, the significance of the interactions formed by Leu283 has been highlighted by a mutation study conducted by Niu et al. [7]. In this study, an L283M mutation led to a reduced potency of SGLT2 inhibition by empagliflozin, indicating the importance of Leu283 for isoform-selective SGLT2 inhibition [7].

**Figure 9:**
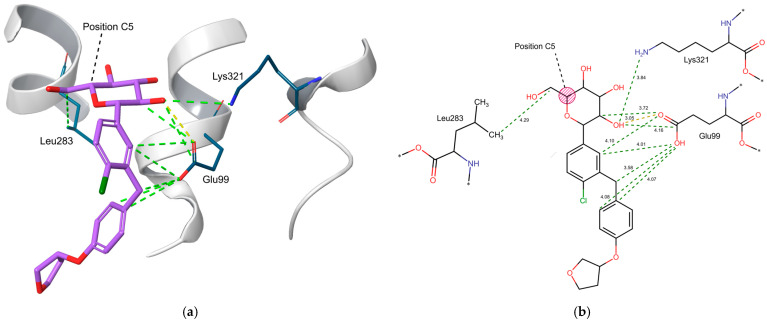
(**a**) 3D representation of the interactions formed by empagliflozin (purple), with residue Glu99 forming a hydrogen bond interaction with the hydroxy group at C2 of the sugar moiety (yellow dotted line) and various positively contributing interactions with the sugar moiety as well as the aglycon group (green dotted lines). Additionally, Leu283 forms an interaction with the CH2OH group at position C5, and Lys321 interacts with the hydroxy group at position C2 (green dotted lines). (**b**) 2D representation of the above-mentioned interactions formed by empagliflozin with Glu99, Leu283, and Lys321 together with the distances of the interactions in Å. The remaining amino acids of SGLT2 connected to Glu99, Leu283, and Lys321 have been denoted with asterisks.

The negatively contributing three-dimensional properties of the *S*-configuration at the sugar moiety—making it harder to achieve a hydrogen bond with Glu99—serve, therefore, as an explanation of the loss of activity. An example of the interactions of empagliflozin, which shows an *R*-configuration at position C5, with the amino acids Glu99, Leu283, and Lys321 is shown in Figure 9. While the amino acid Leu283 directly forms interactions with the CH2OH group at position C5 of the sugar moiety, Glu99 and Lys231 show interactions with the hydroxy group at position C2, with Glu99 forming additional interactions with the two aromatic rings of the aglycon group. The importance of the interaction formed between Glu99 and the hydroxy group of position C2 is further supported by experimental data for molecules with modifications at position C2, where the replacement of the hydroxy group with other functional groups can lead to a complete loss of activity [5].

## 3. Materials and Methods

### 3.1. Data Retrieval

Out of the six available protein structures of human SGLT2, five structures with an outward-open conformation were retrieved from the Protein Data Bank (PDB), which is a publicly available data bank containing information about the 3D structures of proteins, nucleic acids, and complex assemblies [30]. All of the outward-open structures have been elucidated by cryogenic electron microscopy and show a resolution of 3.10 Å or better. The retrieved structures comprise PDB codes 7VSI, 8HEZ, 8HG7, 8HB0, and 8HDH, each of them with an SGLT2 inhibitor at their binding site [7,8] (Table 5). The sixth structure (PDB code 8HIN), which has been recently elucidated in an inward-open conformation with phlorizin bound to it, has not been used for this study due to its unique conformation among the available SGLT2 structures. In contrast to phlorizin, the gliflozins used in the cryo-electron microscopy study presented by Hiraizumi et al. [8] bind to the transporter exclusively in its outward conformation. This is due to the direct connection of the central aromatic ring to the glucose moiety, causing a rigidity that prevents binding to the inward-open structure. Additionally, among the known SGLT2 inhibitors, only phlorizin has been reported to cause weak intracellular inhibition of SGLT2 when using high concentrations in the absence of a Na^+^ gradient [8,31]. Considering the aim of this work being to provide a methodology for a docking-based classification of potential SGLT2 inhibitors, 8HIN was thus determined to be unsuitable for the presented study.

In order to obtain ligands of SGLT2 with known activity, an in-house KNIME (v. 4.4.4, KNIME AG, Zürich, Switzerland) workflow was used which was initially created by members of the Pharmacoinformatics Research Group of the University of Vienna for the retrieval and standardisation of molecules from ChEMBL (v. 30, European Bioinformatics Institute (EBI), Cambridge, UK) and internal datasets to prepare them for machine learning tasks. The workflow was adapted to retrieve and prepare molecules with known activity in an appropriate condition for docking [32].

The KNIME workflow provides a simple way to define the desired target protein and choose a threshold of the pChEMBL activity value. Compounds with activities above this threshold are labelled as active by adding a column which holds the value 1 (active) or 0 (inactive). The desired target protein is chosen by uploading an Excel file containing its ChEMBL ID to the workflow, after which the compounds with missing pChEMBL values, activity values that are 0, and values that are not in an IC50 or Ki unit are excluded. Initially, the newest ChEMBL database version available for this workflow was ChEMBL 29. Therefore, the workflow had to be adapted in order to allow the use of the newest version of ChEMBL, which was ChEMBL 30 at the time of this project [33]. In the following section of the workflow, there is an option to standardise the retrieved compounds. The settings of the node responsible for the standardisation process were changed to achieve an appropriate output for docking studies: the stereochemistry was not removed, and molecules with nonorganic atoms were kept. The following step is characterised by the addition of the activity classification (0 or 1) based on the chosen threshold for the activity values. During this step, molecules with multiple activities are merged into one entry and filtered depending on the properties of all the activity values: If all activity values lead to the same activity classification, the compound does not become excluded. If some activity values lead to different activity classifications, the compound is filtered out. However, after docking, all molecules with multiple activity values had to be removed in order to allow for consistency among the classification models. Afterwards, the retrieved molecules are saved as a file in the SDF format as part of the workflow output, which then may be further used for docking and machine learning purposes.

1197 ligands were retrieved from the ChEMBL database with activities ranging from pChEMBL values of 4 to slightly above 10. An analysis of the SGLT2 inhibitors currently approved for therapeutic use reveals that the majority of compounds have an IC50 value corresponding to pChEMBL values between 8 and 9 [34,35,36]. As activity thresholds are usually set at a micromolar or even nanomolar range, and the assessment of virtual screening protocols necessitates a reasonable quantitative relationship between actives and inactives, the classification threshold was set at pChEMBL = 8 [37,38].

### 3.2. Molecular Docking

All docking protocols were performed in Maestro (Schrödinger Release 2021-1: Maestro, Schrödinger, LLC, New York, NY, USA).

As it was shown that the negligence of certain preparation steps in molecular docking studies may lead to significant drops in the performance of virtual screening studies, it is generally agreed upon to properly prepare the protein crystal structure as well as the ligands for molecular docking [39]. The Protein Preparation Wizard was employed to preprocess the protein structures. As an addition to the default settings, missing side chains and loops were filled, and the termini were capped. The ligand preparation was conducted as a part of the Virtual Screening Workflow, which was also employed to perform the docking tasks. The ligand preparation was run using default settings of LigPrep with the exception of an additional desalting of the ligands, which is a necessary step in the docking process [40]. Docking was performed with the standard precision mode (Glide SP) of the Virtual Screening Workflow, which is a semi-rigid docking algorithm. Semi-rigid docking methods—where the ligand is considered flexible, but the protein is not—are a suitable approach for the screening of large ligand libraries [41], which is the reason it has been chosen for this study. Even though Glide provides two additional scoring functions, which take advantage of the semi-rigid docking method, Glide SP was identified as the ideal choice for the development of the methods of this study, as it is computationally less expensive than the Glide XP scoring function, and therefore better suited for screening tasks while maintaining a higher accuracy than the Glide HTVS scoring function [42].

For the docking process provided by the Virtual Screening Workflow, all default settings were kept, with the exception of retaining all successfully docked molecules.

The results from docking into all five human SGLT2 structures were compared. The ability of the docking algorithm to correctly reproduce the binding mode of SGLT2 inhibitors was evaluated by redocking their ligands and visually assessing the poses. The performance of the docking protocols was assessed by screening the retrieved library of compounds with known activity and calculating the 10% EF and the ROC AUC using the Enrichment Calculator of the Schrödinger software [43].

### 3.3. Classification Models

The following investigations have been conducted using Python (v. 3.10.13). All classification models for potential SGLT2 inhibitors were created by using a Kernel Density Estimation (KDE), which is, as described in the documentation of the seaborn package [44], a method for visualising the distribution of observations in a dataset, analogous to a histogram. In a KDE plot, the data are represented by using a continuous probability density curve in one or more dimensions. The KDE of the two distribution functions of the active and inactive molecules were plotted by using the Matplotlib package (v. 3.8.2) in Python and calculating their intersection. The resulting intersection was used as a threshold for classifying molecules as active or inactive [37,45].

The choice of a docking score threshold for a predicted classification of ligands into actives and inactives is a context-dependent process and has to take into account practical considerations of the investigator [45,46]. Nevertheless, using the kernel density estimation allows the calculation of a reasonable threshold by finding the intersection point of the two distribution functions. Choosing this intersection point leads to a classification threshold which minimises both false positives and false negatives equally. This constitutes a liberal strategy that allows taking the uncertainty of the model into account while losing fewer actives in a virtual screening task compared to strategies where minimising false positives is prioritised [45]. For the sake of convenience, this method has been called the “Threshold Method” throughout this article.

In order to express the performances of the created classification models, the Matthew’s Correlation Coefficient (MCC) as well as the Balanced Accuracy (BA) has been used. The MCC is a suitable performance metric for imbalanced datasets in binary classification tasks and possesses the key advantage of generating a high score only if there is a high percentage of correct predictions for both negative and positive data instances. It ranges between −1 and +1, with higher scores indicating better performance [47].

$$MCC\;=\;\frac{\mathrm{TP}\times \mathrm{TN}-\mathrm{FP}\times \mathrm{FN}}{\sqrt{\left(\;\left(\mathrm{TP}\;+\;\mathrm{FP}\right)\times \left(\mathrm{TP}\;+\;\mathrm{FN}\right)\times \left(\mathrm{TN}\;+\;\mathrm{FP}\right)\times \left(\mathrm{TN}\;+\;\mathrm{FN}\right)\right)}}$$

where *TP*, *TN*, *FP*, and *FN* represent True Positives, True Negatives, False Positives, and False Negatives, respectively. As shown, the *MCC* is proportional with true positive and true negative calls, while it is lower when more false positive and false negative calls are obtained. As an additional metric, the better-known balanced accuracy has been used to make an easier interpretation of the obtained results possible.

$$BA\;=\frac{1}{2}\times \left(\frac{TP}{TP+FN}+\frac{TN}{TN+FP}\right)$$

Similarly to the *MCC*, the *BA*, which ranges between 0 and 1, is not sensitive to dataset imbalances, and a higher *BA* indicates a better performance [48].

#### 3.3.1. Docking Score-Based Classification Model

Based on the docking scores obtained from docking into all five human SGLT2 structures and their performances in the validation step, a classification model for potential SGLT2 inhibitors was created by using the best-performing SGLT2 structure. For this purpose, the above-mentioned threshold method was used to obtain a threshold for classifying molecules as active or inactive [37,45].

#### 3.3.2. Ensemble Docking-Based Classification Models

In addition to the docking score-based classification model, the possible improvement of docking scores utilising ensemble docking was explored. These ensemble docking scores were obtained by calculating the mean score of each molecule in concordance with the rank-by-number strategy described by Wang and Wang [49]. This method calculates the arithmetic mean of the docking scores of each molecule originating from docking into the SGLT2 structures, which was conducted for every possible number of combined targets (2–5) in every possible combination. Afterwards, the mean scores were used to create classification models by applying the above-mentioned threshold method.

#### 3.3.3. Structural Similarity-Based Classification Models

Finally, the methodology of using structural similarity in order to enhance scoring functions, put forward by Ji et al. [26], was expanded upon and used to further improve the classification models. The similarity-enhanced scores were then used to create classification models by applying the above-mentioned methodology of calculating the intersection points of active and inactive molecules.

The methodology uses a correction algorithm for the docking scores by utilising structural similarity and the distance of the docking scores from the known activities of the molecules. Here, the Tanimoto similarity of each molecule from a query dataset, for which an improvement of the docking scores is desired, to each molecule of a reference dataset with known binding affinities and docking scores is calculated. The distances between the docking scores and the actual binding affinities of the training set molecules weighted by the above-mentioned similarities are then used to calibrate the docking scores of the query dataset. Therefore, a molecule from the reference dataset with high similarity to a query molecule will have a higher impact on the calibration of the query molecule than a molecule with low similarity.

$$DS_{j}=DS_{j}^{0}\left[\frac{1}{\omega }\sum_{i\neq j}^{n}S_{ij}^{p}\frac{\Delta G_{i}}{DS_{i}}\right]\;\omega =\sum_{i\neq j}^{n}S_{ij}^{p}$$

Referencing the authors of the above-mentioned work, the algorithm is described as follows:

“where $DS_{j}^{0}$ and $DS_{j}$ are the docking score of the *j*th query compound before and after the calibration. $S_{ij}$ is the structural similarity between the *j*th query compound and the *i*th reference ligand. The exponent *p* is treated as an integer constant with its value varying from 1 to 4 in this study, for the exploration of the developed formula. We referred $S_{ij}^{p}$ as compound similarity effect (CSE) function for convenience of discussion. n is the total number of reference ligands in the reference dataset. $DS_{i}$ is the docking score of the *i*th reference ligand. $\Delta G_{i}$ is the experimental binding energy (kcal/mol) of the *i*th compound in the reference dataset […].”

[26].

Even though a brief explanation is provided for the usage of exponent *p* with a value ranging from 1 to 4, a further exploration of this concept using values ranging from 4 to 60 in steps of 8 allowed us to investigate the relationship between the importance given to the structural similarity (higher importance for higher values of exponent *p*) and the improvement of the docking scores. By exploring a wider range of values of exponent *p* in a grid search and alternative fingerprint algorithms, the methodology was further improved for our use case.

The used fingerprints for determining the structural similarity were the MACCS keys, the Morgan fingerprint, and the RDKit fingerprint, which were calculated using RDKit (v. 2023.9.4). The MACCS (Molecular ACCess System) keys is a structural keys fingerprint, which encodes the structure of a molecule into a binary bit string, where each bit is representing one key out of a list of 166 structural fragments [50]. The Morgan fingerprints provided by RDKit, which are also known as circular fingerprints, are generated by considering the environment of the atoms in a molecule up to a certain radius. The radius and the atom identifiers, which were used for the calculation of the Morgan fingerprint, were set to their default values of the RDKit package [51]. Finally, the third fingerprint type used in this study, which is the RDKit fingerprint, is a path-based fingerprint. Path-based fingerprints are calculated by generating fragments of the molecule by following a path up to a certain number of bonds within the molecule, thereby creating a list of every pattern within the molecule up to the predefined length [52].

This methodology was used to further improve the classification models, using the resulting similarity-enhanced scores to create models by applying the above-mentioned threshold method. The final model from each fingerprint methodology was then applied to an external dataset to gauge their performance.

## 4. Conclusions

The presented work has succeeded in its objective to provide classification models for the prediction of SGLT2 inhibitors that incorporate three-dimensional information. In order to achieve this aim, three different methods to create docking score-based classification models have been explored. Creating models by combining structural similarity and docking scores into one classification model proved to be the best-performing method, with the best model achieving an MCC of 0.64, while ensemble docking-based models outperformed the direct usage of docking scores coming from one docking study. Furthermore, using docking studies to predict activities of molecules which only differ in their configuration proved to be a promising approach, allowing the discovery of lost ligand–protein interactions for inactive configurations. This approach proved Glu99 to be the most important interaction loss for SGLT2 inhibitors with an *S*-configuration at position C5. With this study, we demonstrate that the usage of three-dimensionally aware classification models represents a versatile method for modelling the inhibition of targets sensitive to the configuration of their ligands.

## Figures and Tables

**Table 1:** Results of the virtual screening validation. 7VSI clearly displayed the best performance using both performance metrics, while 8HB0 and 8HDH showed the worst performance.

Protein (PDB ID)	ROC AUC	10% EF	Number of Docked Ligands	Number and Percentage of Ligands Not Docked
7VSI	0.72	1.5	1147	50 (4.18%)
8HEZ	0.66	1.5	1120	77 (6.43%)
8HG7	0.67	1.4	1158	39 (3.26%)
8HB0	0.59	1.2	1112	85 (7.10%)
8HDH	0.60	1.1	1157	39 (3.34%)

**Table 2:** Performance of the best-performing ensemble docking-based classification model for each combinatory length. The best performance was achieved for the combination of 7VSI, 8HEZ, and 8HG7 with an MCC of 0.41.

Metrics	7VSI, 8HG7	7VSI, 8HEZ, 8HG7	7VSI, 8HEZ, 8HG7, 8HB0	7VSI, 8HEZ, 8HG7, 8HB0, 8HDH
Balanced Accuracy	0.70	0.71	0.69	0.68
MCC	0.39	0.41	0.38	0.36

**Table 3:** Performances, exponent *p*-values, and average intersection points of the best models from each fingerprint method.

	Morgan Fingerprint	RDKit Fingerprint	MACCS Keys
MCC—cross-validation	0.64	0.60	0.61
MCC—external dataset	0.63	0.58	0.67
Exponent *p*	12	28	44
Average intersection point	7.82	7.82	8.04

**Table 4:** Performances of the best models from each of the three studied methodologies using the MCC metric. The structural similarity-based model using the Morgan fingerprint and an exponent *p*-value of 12 clearly outperforms the other two models.

	Structural Similarity Morgan Fingerprint	Ensemble Docking 7VSI, 8HEZ, 8HG7	Docking Score 7VSI
MCC	0.64	0.41	0.36

**Table 5:** Retrieved structures, their resolutions, and the ligands they have been resolved with.

Protein (PDB ID)	Resolution (Å)	Ligand
7VSI	2.95	Empagliflozin
8HEZ	2.80	Dapagliflozin
8HG7	3.10	Sotagliflozin
8HB0	2.90	TA1887
8HDH	3.10	Canagliflozin

## Data Availability

The original data presented in the study are openly available in PHAIDRA at https://phaidra.univie.ac.at/o:2112304 (accessed on 13 May 2025).

## Supplementary Materials

The following supporting information can be downloaded at: https://www.mdpi.com/article/10.3390/molecules30102179/s1. Table S1: Performance of each of the Consensus Score based classification models for each combinatory length of the five SGLT2 structures using the Matthews Correlation Coefficient (MCC) as the performance metric. Table S2: Matthews Correlation Coefficient of RDKit fingerprint, Morgan fingerprint, and MACCS keys models for *p*-values ranging from 4 to 60 in steps of 8. Figure S1: Redocked- (orange) and cryo-EM (purple) poses of the ligands bound to SGLT2 structures (a) 7VSI, (b) 8HEZ, (c) 8HG7, (d) 8HB0, (e) 8HDH.
