# Supplementary material for: Docking-Based Classification of SGLT2 Inhibitors
Source: Molecules. 2025 May 16;30(10):2179. doi: 10.3390/molecules30102179 (PMC12114233; doi:10.3390/molecules30102179)
Supplement: Supplementary file 1 [file molecules-30-02179-s001.zip › molecules-3616477-supplementary.pdf]

Supporting information

Table S1. Performance of each of the Consensus Score based classification models for each combinatory length of the five SGLT2 structures using the Matthews Correlation Coefficient (MCC) as the performance metric. A combination of 7VSI, 8HEZ and 8HG7 showed the best performance while the combination of 8HB0 and 8HDH performed the worst.

	Combinations (PDB IDs)	MCC
Combinations of 2	7VSI, 8HEZ	0.37
	**7VSI, 8HG7**	**0.39**
	7VSI8, HB0	0.35
	7VSI, 8HDH	0.31
	8HEZ. 8HG7	0.38
	8HEZ, 8HB0	0.32
	8HEZ, 8HDH	0.30
	8HG7, 8HB0	0.34
	8HG7, 8HDH	0.32
	8HB0, 8HDH	0.26
Combinations of 3	**7VSI, 8HEZ, 8HG7**	**0.41**
	7VSI, 8HEZ, 8HB0	0.38
	7VSI, 8HEZ, 8HDH	0.34
	7VSI, 8HG7, 8HB0	0.36
	7VSI, 8HG7, 8HDH	0.33
	7VSI, 8HB0, 8HDH	0.31
	8HEZ, 8HG7, 8HB0	0.36
	8HEZ, 8HG7, 8HDH	0.33
	8HEZ, 8HB0, 8HDH	0.28
	8HG7, 8HB0, 8HDH	0.31
Combinations of 4	**7VSI, 8HEZ, 8HG7, 8HB0**	**0.38**
	7VSI, 8HEZ, 8HG7, 8HDH	0.37
	7VSI, 8HEZ, 8HB0, 8HDH	0.34
	7VSI, 8HG7, 8HB0, 8HDH	0.34
	8HEZ, 8HG7, 8HB0, 8HDH	0.34
Combination of 5	**7VSI, 8HEZ, 8HG7, 8HB0, 8HDH**	**0.36**

Table S2. Matthews Correlation Coefficient of RDKit fingerprint, Morgan fingerprint, and MACCS keys models for p-values ranging from 4 to 60 in steps of 8. The fingerprints showed their best performance at p-values of 28, 12, and 44, with the Morgan fingerprint achieving the best performance among all fingerprints with a MCC of 0.642. In order to represent the subtle performance differences, MCC values are shown with three decimal places.

	**Exponent p**	**MCC**
RDKit fingerprint	4	0.467
	12	0.594
	20	0.602
	**28**	**0.604**
	36	0.601
	44	0.599
	52	0.596
	60	0.601
Morgan fingerprint	4	0.520
	**12**	**0.642**
	20	0.638
	28	0.634
	36	0.637
	44	0.628
	52	0.624
	60	0.627
MACCS keys	4	0.446
	12	0.571
	20	0.610
	28	0.609
	36	0.599
	**44**	**0.612**
	52	0.608
	60	0.608

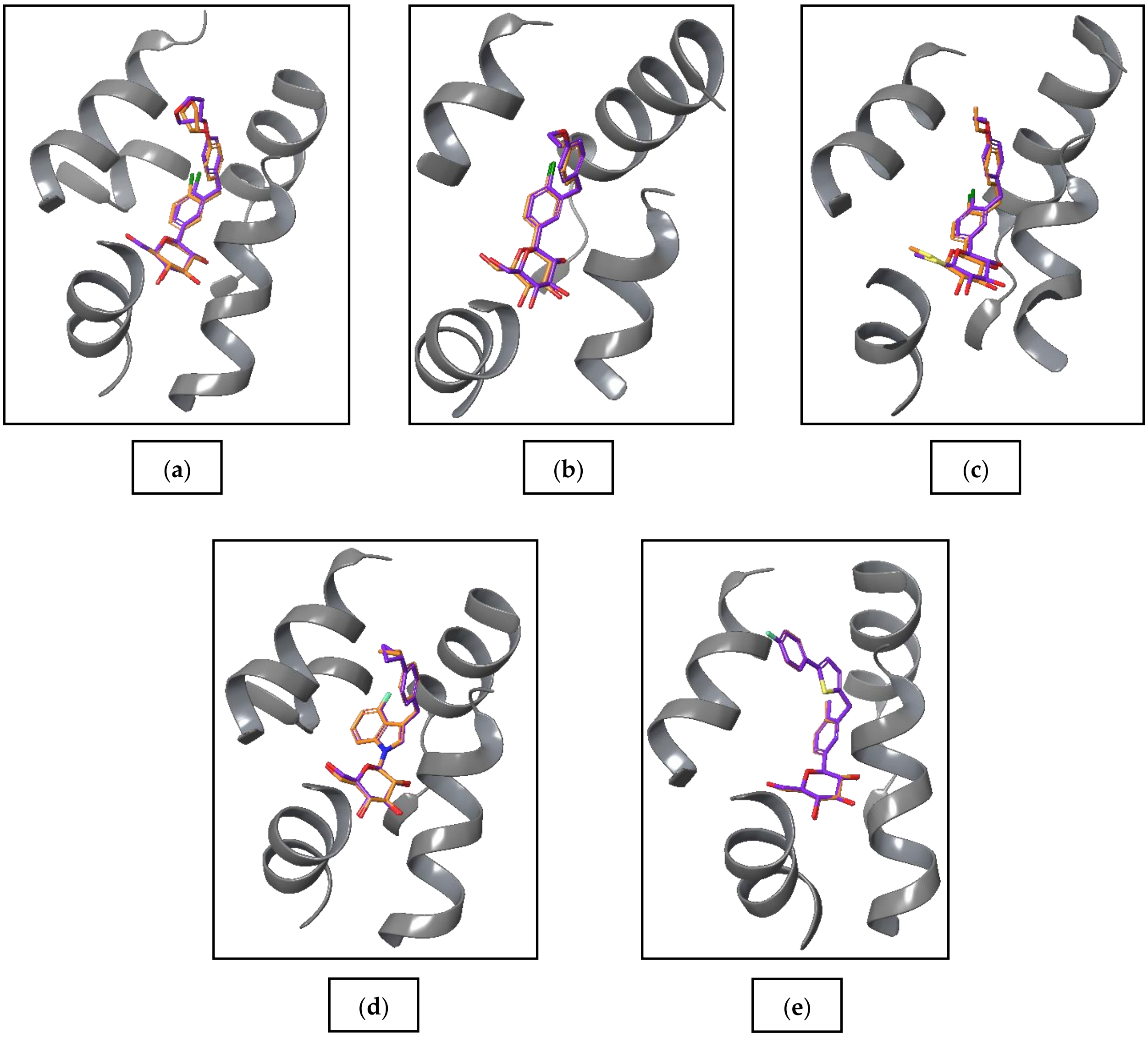

Figure S1. Redocked- (orange) and cryo-EM (purple) poses of the ligands bound to SGLT2 structures (**a)** 7VSI, (**b**) 8HEZ, (**c**) 8HG7, (**d**) 8HB0, (**e**) 8HDH. After redocking, the poses of each ligand were sorted using the Glide Emodel Score, which is suited for comparing conformers, and the top poses were chosen for comparison to the cryo-EM poses.
